# Brainmask: an ultrasoft and moist micro-electrocorticography electrode for accurate positioning and long-lasting recordings

**DOI:** 10.1038/s41378-023-00597-x

**Published:** 2023-10-10

**Authors:** Bowen Ji, Fanqi Sun, Jiecheng Guo, Yuhao Zhou, Xiaoli You, Ye Fan, Longchun Wang, Mengfei Xu, Wen Zeng, Jingquan Liu, Minghao Wang, Huijing Hu, Honglong Chang

**Affiliations:** 1 Unmanned System Research Institute, Northwestern Polytechnical University, Xi’an, 710072 China; 2 Ministry of Education Key Laboratory of Micro and Nano Systems for Aerospace, School of Mechanical Engineering, Northwestern Polytechnical University, Xi’an, 710072 China; 3 Collaborative Innovation Center of Northwestern Polytechnical University, Shanghai, 201108 China; 4 Institute of Medical Research, Northwestern Polytechnical University, Xi’an, 710072 China; 5 College of Electronics and Information, Hangzhou Dianzi University, Hangzhou, 310018 China; 6 National Key Laboratory of Science and Technology on Micro/Nano Fabrication, Department of Micro/Nano Electronics, Shanghai Jiao Tong University, Shanghai, 200240 China

**Keywords:** Electrical and electronic engineering, Electronic devices, Structural properties

## Abstract

Bacterial cellulose (BC), a natural biomaterial synthesized by bacteria, has a unique structure of a cellulose nanofiber-weaved three-dimensional reticulated network. BC films can be ultrasoft with sufficient mechanical strength, strong water absorption and moisture retention and have been widely used in facial masks. These films have the potential to be applied to implantable neural interfaces due to their conformality and moisture, which are two critical issues for traditional polymer or silicone electrodes. In this work, we propose a micro-electrocorticography (micro-ECoG) electrode named “Brainmask”, which comprises a BC film as the substrate and separated multichannel parylene-C microelectrodes bonded on the top surface. Brainmask can not only guarantee the precise position of microelectrode sites attached to any nonplanar epidural surface but also improve the long-lasting signal quality during acute implantation with an exposed cranial window for at least one hour, as well as the in vivo recording validated for one week. This novel ultrasoft and moist device stands as a next-generation neural interface regardless of complex surface or time of duration.

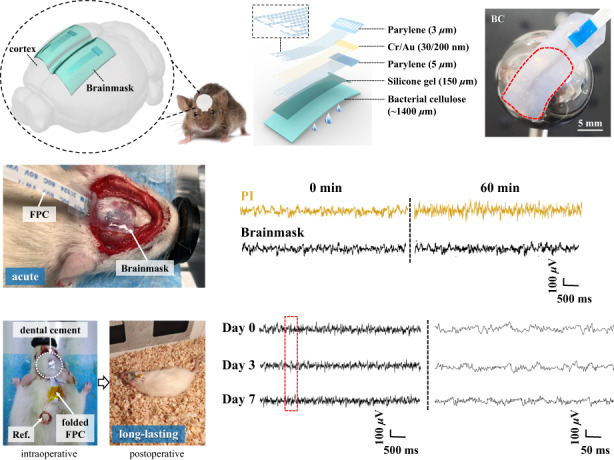

## Introduction

Implantable neural interfacing electrodes serve as the basic research tools for neuroscience^1–4^ as well as the clinical application tools for the diagnosis and treatment of neurological disorders^5–9^, thanks to the functions of electrophysiological recording and/or neural stimulation or modulation. Electrocorticography (ECoG) records the electrical activity in the brain from the sum of the local field potentials of the population of neurons by flexible electrodes implanted on the epidural or subdural surface of the brain^10^. In the last decade, minimally invasive ECoG electrodes have no longer been limited to short-term neural signal monitoring and intraoperative focal localization in surgery for epilepsy^11–13^. More attention has been given to long-term implants for the early prediction and timely prevention of seizures, such as the FDA-approved closed-loop responsive neurostimulator (RNS® System; NeuroPace, Inc., Mountain View, CA, United States)^7,14^, and recent reports on applications to decode motor, vision, or speech^15–17^. For chronic ECoG electrodes, continuous micromotion, such as changes in blood flow, respiration, movement, and intracranial pressure, will cause relative displacement between the electrodes and soft brain tissue^18,19^. Meanwhile, the mechanical mismatch between the electrodes and brain can easily cause foreign body responses^19–22^, which will further degrade the signal quality and hinder stable usage in the long term. Therefore, the electrodes in close contact with the soft brain tissue should be in a softer physical state^23–25^.

Currently, most commercially available ECoG cortical electrodes are the macro-electrodes composed of silicone and platinum-iridium alloy^26,27^, such as conventional ECoG devices from Cortec GmbH, g.tec medical engineering GmbH, PMT Corporation, Ad-Tech Medical Instrument Corporation, etc. The relatively large thickness of the macro-electrodes leads to insufficient conformation of the superficial sulci and gyri of the brain. As potential alternatives, thin-film surface microelectrodes made of polymer materials such as polyimide, parylene-C or SU-8 possess higher channel counts and resolution^28,29^. In addition, the extremely low substrate thickness of these electrodes can effectively improve the performance of its conformal contact with soft brain tissue^30^, but its mechanical strength is insufficient, and no product has yet been approved for clinical use. Only the Evo® Cortical Electrode (NeuroOne Medical Technologies Corporation) with an 80-micron-thick polyimide substrate is allowed to be implanted in humans for 30 days.

A significant research trend is how to further reduce the stiffness of microelectrodes. One method is to improve the deformation adaptability of the electrode itself by structural design, such as the employment of a segmented discrete structure. A mesh structure can ensure the relative spatial position of the electrode sites, but the interconnects constrain these sites from each other^30^. A strip structure reduces the mutual constraints between electrode sites, but it increases the operation difficulty in the implantation process simultaneously^31^. A kirigami structure has good stretchability, but the electrode sites will undergo out-of-plane deformation with the stretched substrate, which induces poor adhesion to the tissue surface^32^. Another method is to use intrinsically soft materials, which have similar mechanical properties to soft neural tissue, to help reduce the micromotion between electrodes and the brain, improve conformability, and ensure signal quality during long-term implantation^19^. Softer silicone materials such as polydimethylsiloxane (PDMS) have been directly applied as substrates^33–37^, but issues with the uneasy patterning of PDMS still exist, as well as the weak bonding and rupture risk of conductive layers such as gold films encapsulated in PDMS. In other studies, parylene-C films have been deposited on the surface of PDMS to fabricate ECoG electrodes^23,38^, or integrated polymer thin-film electrodes with a serpentine structure have been printed on the surface of elastomers by transfer printing^39,40^. In addition, some researchers have used hydrogels as substrates, which are softer than PDMS^41–43^, but the poor processing accuracy and low spatial resolution limit their application. In addition, its biocompatibility and long-term reliability have yet to be verified.

Another problem that can be easily overlooked is the moisturizing properties of electrodes. The brain cavity is a relatively closed environment surrounded by cerebrospinal fluid, providing the brain with complete sterility, suitable temperature and pressure, adequate nutrition, and a safe environment. During electrode implantation, the contact interface between the cortical electrodes made of silicone or polymer substrate and the brain tissue gradually dries with the evaporation and loss of cerebrospinal fluid, which results in a decline in signal quality^43,44^. Meanwhile, the subdural cortex may stick to the electrodes without continuous protection from the cerebrospinal fluid, which is easily affected by external forces (e.g., pulling at the end of the cable), resulting in electrode damage or tissue bleeding. However, if artificial cerebrospinal fluid (ACSF) is dripped during the operation to keep the brain tissue in a moist state, then the electrode may float and thus separate from the brain surface. If a brain cotton slice or gelatin sponge that absorbs ACSF is used to cover the electrode to keep it moist, then the electrode itself will be blocked^44^, which is not beneficial for observing whether the electrode has a small displacement or subdural hematoma formation. In most studies, ECoG electrodes are used in acute experiments to verify in vivo functions and localize focal epileptic foci. During the operation, a craniotomy was performed with the dura removed to expose the target brain area, and ACSF was needed to keep the site moist, stop bleeding and maintain clear vision^45^. In addition, although hydrogels have a certain moisture-retaining capacity, their processing accuracy is insufficient, as mentioned above; this is an obstacle for the development of miniaturized and high-density electrodes^41,43^.

To guarantee conformal contact between micro-ECoG electrodes and brain tissue and protect the brain tissue by lasting intraoperative moisturizing, we chose bacterial cellulose (BC) film as the substrate material for the electrodes. Bacterial cellulose is a highly biocompatible material with an ultrafine mesh structure, and the film made from this material has good softness and strong water absorption and moisture retention^46^ and has been widely used in moisturizing facial masks^47^. The Young’s modulus of the BC film can be as low as 80–120 kPa after water absorption and expansion, which is much lower than that of polyimide and PDMS. Its softness ensures close adhesion to the wrinkled outer surface of the brain^48^. In addition, the quality of the BC film in the fully absorbent state is approximately 100 times that in the dry state^49^. This indicates that the BC film can store a large amount of ACSF and help press the electrode close to the brain tissue by its own weight, which can improve the contact effect and then the signal quality. However, there are potential problems for BC films as electrode substrates, including poor insulation, low compatibility with micro/nano manufacturing and insufficient durability. Therefore, new technical solutions need to be considered to ensure that the conductive materials are in good packaging condition when combined with the BC film.

**Fig. 1:**
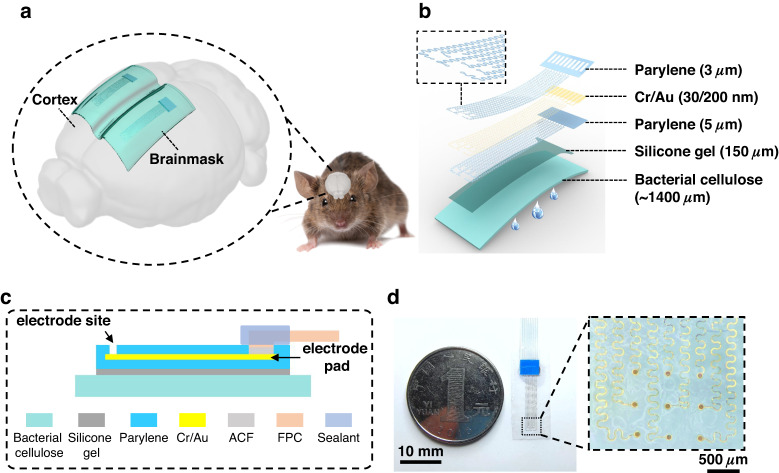
Overview of the Brainmask device. **a** Schematic illustration of the conformable device on the curved rodent brain. **b** Explosive view of the device with ultrasoft and moist bacterial cellulose as the substrate. **c** Cross-sectional view of the device with the electrode sites and pads exposed from the parylene-C thin film. **d** Picture of the device consisting of 9 independent electrode channels with diameters of 100 *µ*m and pitches of 700 *µ*m, as well as serpentine interconnects

In this work, for the first time to our knowledge, an innovative micro-ECoG electrode composed of a layer of bacterial cellulose (BC) substrate and a metal layer sandwiched between two parylene-C layers with a serpentine layout is proposed (Fig. 1b, c). This structure gives full play to the ultra-softness and high moisture retention of the BC film, which ensures the close contact of the electrodes to the brain and maintains moisture (Fig. 1a). Because it is similar to a commercial facial mask, we name it “Brainmask”. The water-tight and biocompatible serpentine parylene-C microelectrodes can be bonded with the BC film, as pictured in Fig. 1d. In this way, this mask can not only ensure the accurate positioning of microelectrode sites but also improve long-lasting signal quality during acute implantation with an exposed cranial window, as well as long-lasting in vivo recordings for one week.

## Results

### Accurate positioning of the serpentine electrode with the BC substrate

**Fig. 2:**
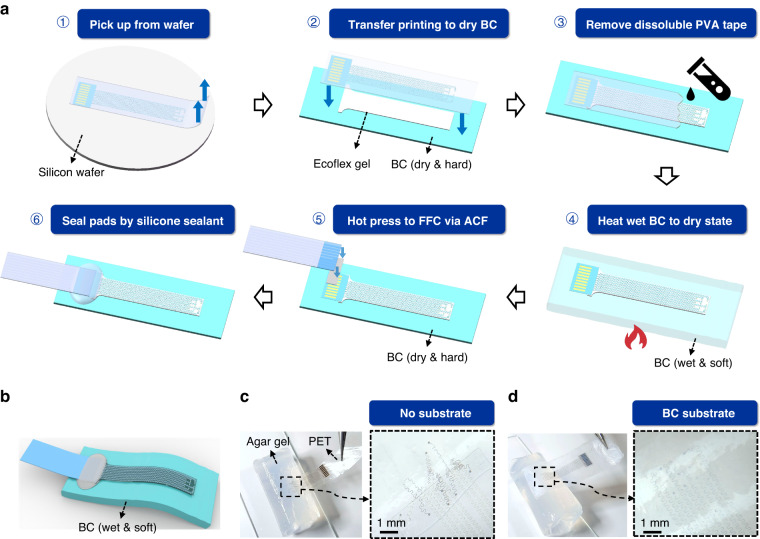
Assembly of the Brainmask device with accurate positioning. **a** Assembly processes of the serpentine electrode to the BC film by transfer printing and ACF hot pressing. **b** Schematic diagram of the final state of the ultrasoft and moist Brainmask. Pictures of (**c**) disordered serpentine electrode with no substrate and (**d**) accurately positioned serpentine electrode with the BC substrate attached to the 0.6% agar gel cylinder (5 mm in radius)

The assembly processes were developed based on the water loss and absorption characteristics of the BC film to ensure accurate positioning. The preparation of the 9-channel Brainmask device is shown in Fig. 2a. First, polyvinyl alcohol (PVA) tape (ASWT-1, AQUASOL, USA) was used to pick up the serpentine electrode from the silicon wafer, and the relative position between the interconnects remained unchanged. The BC film was dried at room temperature, followed by coating with a thin layer of elastic silica gel (33.4 kPa, Ecoflex Gel, Smooth-on, USA)^50^ as an intermediate adhesive layer. Then, the serpentine electrode was quickly attached on the top surface of the elastic silica gel layer. Notably, no gel is brushed under the local area of the electrode pads to ensure its relatively high hardness for subsequent hot pressing. Next, the sample was slowly rinsed with deionized water at 40 °C to completely dissolve the PVA tape, and the BC film absorbed water and swelled sequentially. The device was heated in an air oven until the wet BC film evaporated to the dry state. Finally, the pad area was hot pressed to a flexible flat cable (FFC) via an anisotropic conductive film (ACF, AC2056R, HITACHI, Japan), followed by sealing the interface with silicone sealant (K-5905L, Kafuter, China). As illustrated in Fig. 2b, the final Brainmask device was restored to the wet and soft state after assembly by absorbing water again.

The MEMS-fabricated micro-ECoG electrodes based on parylene-C (as shown in Supplementary Fig. S2) or polyimide thin film (as shown in Supplementary Fig. S3) employ the fractal serpentine design to ensure conformality to the curved brain surface. However, the individual channels of the released electrode are easily tangled, as we have previously reported^40^. Therefore, transfer printing by retrieval via water-soluble adhesive tape from the silicon wafer provides a solution to guarantee relative position accuracy. During the transfer process, a small area of gel under the front edge region (0.5 mm in length) of the rectangular pad region was retained (Supplementary Fig. S4), which not only guaranteed sufficient mechanical strength to avoid failure of adjacent serpentine interconnects but also facilitated the following hot-pressing step and implantation. To further explain the necessity and advantage of the BC film as an ultrasoft substrate of the Brainmask, the serpentine electrodes with no substrate and BC substrate were separately attached to a 0.6% agar gel cylinder with a radius of 5 mm, as shown in Fig. 2c, d. The radius of curvature of a rat brain is approximately 3 mm^22^, similar to that of a agar gel cylinder, demonstrating conformal attachment and accurate positioning (Supplementary Fig. S5). The attachment processes of the serpentine electrodes without and with BC substrate can be seen in Supplementary Movies 1 and 2. Thus, the interface between the serpentine electrode and BC substrate, with the aid of an ultrathin, ultrasoft and sticky layer of Ecoflex gel, ensures the accuracy of the relative position of all electrode channels and avoids fracture or worse failure.

### Ultrasoftness of the BC film as a substrate

**Fig. 3:**
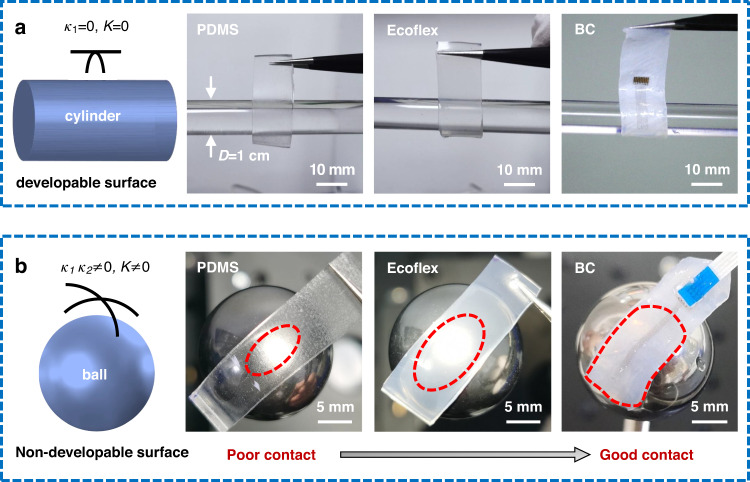
Comparison of different substrate materials in terms of softness. **a** Wrapping the PDMS, Ecoflex and wet BC samples of the same thickness on the developable surface of the cylinder and (**b**) adhering these samples on the nondevelopable surface of the ball, with a red dashed circle to mark the contact area

To demonstrate the unique advantage of the BC film as a substrate, it was compared with two kinds of commonly used soft silicones, namely, PDMS (base-to-cure ratio of 10:1, *E* = 1.0 MPa) and Ecoflex (A:B = 1:1, *E* = 0.06 MPa), with the same thickness. First, we wrapped the PDMS, Ecoflex and wet BC samples with the same length, width and thickness (approximately 1.4 mm in thickness) on a cylinder with a developable surface (the axial curvature of the cylinder *κ*_*1*_ = 0, the Gaussian curvature *K* = 0), as shown in Fig. 3a. The PDMS sample cannot contact conformally on the cylinder, and the Ecoflex sample can warp better than the PDMS with a slight separation at the bottom edge. In comparison, the wet BC sample can warp best owing to the water capillarity as an adhesion force at the interface and its ultrasoftness. However, the cerebral cortex is a nondevelopable surface. Thus, we attached these three samples on a ball with a nondevelopable surface (the double curvature of the sphere *κ*_*1*_*κ*_*2*_ ≠ 0, the nonzero Gaussian curvature *K* ≠ 0) and marked the contact area, as pictured in Fig. 3b. Compared with the PDMS sample, the wet BC sample maintains good contact, even with the spherical surface.

To further validate the adhesion effect, which mimics the sulci and gyri on the surface of the brain, we used Ecoflex and wet BC films (both approximately 1.4 mm in thickness) to adhere to a large wave board (2.5 mm in radius) and a small wave board (0.7 mm in radius) similar to a row of serial cylinders, smaller than the radius of curvature of the rat brain, as shown in Supplementary Fig. S6. From the top view, many continuous strip gaps or large bubbles appear in the valley for the Ecoflex film, but only a few small bubbles appear with most of the area attached conformally, even in the valley for the wet BC film. The sectional view at one end of the board further exhibits no gap in the valley for BC. In addition, the smaller the radius of the wave board is, the more easily the Ecoflex film can contact the valley of the periodic semicircular board. In comparison, the wet BC film can adhere to either the small or large wave board in good condition. Furthermore, the von Mises stress of the BC film adhered to the wave board (2.5 mm in radius) exhibits a maximum value of 55 kPa, as simulated in the commercial software ABAQUS, which is exhibited in Supplementary Fig. S7. Therefore, the Brainmask device with an ultrasoft BC substrate exhibits distinctly superior performance in terms of attachment to any complex surface; this device can be applied for different experimental animals, including rodents, minipigs, primates, etc.

### Mechanical characteristics

**Fig. 4:**
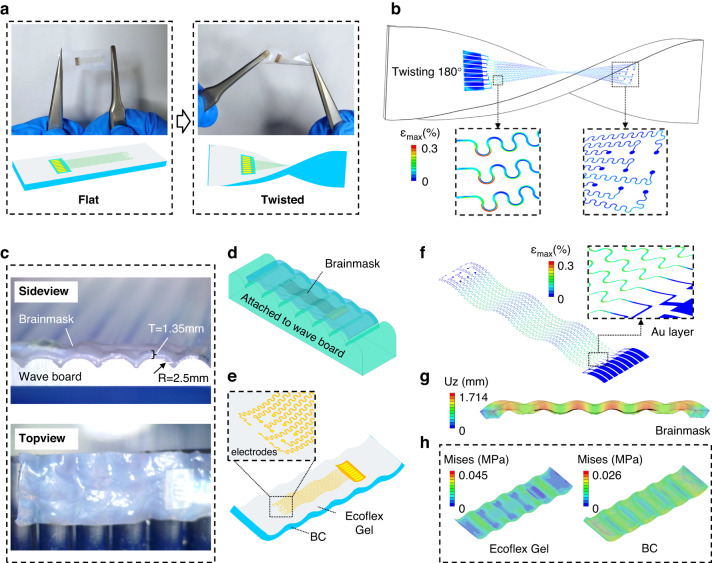
Mechanical characteristics of the Brainmask device. **a** Photos and models of the device when twisted 180°. **b** The FEA strain distribution of the Au metal layer in the device under 180° twisting. **c** Side view and top view photos and (**d**) model of the device attached conformally to the wave board with a radius of 2.5 mm. **e** The other side of the device with electrodes on top of the BC film with the aid of soft Ecoflex Gel. **f** The FEA strain distribution of the Au metal layer, (**g**) the global z-axis displacement of the device, and (**h**) the von Mises stress distribution of the Ecoflex Gel and BC layers when attached to the wave board

The mechanical properties of the Brainmask device were deeply studied by finite element analysis (FEA). Two states of twisting and attaching were simulated in ABAQUS, which allows accurate prediction of the strain or stress distribution and mechanical deformation of the metal layer and substrates. As shown in Fig. 4a, the pictures exhibit flat and twisted 180° states with corresponding models, demonstrating its ability to withstand complex large deformation. The maximum strain of the Au metal layer in the device does not exceed a yield strain of 0.3%, which proves that it is in the elastic state during twisting with the BC substrate (Fig. 4b and Supplementary Movie 3). The serpentine interconnects close to the electrode pads present the largest stress in the whole device but still do not exceed the yield strain of Au (0.3%).

To verify the attachment effect to the sharp curvature variation, we chose a periodic semicircular wave board with a radius of 2.5 mm, as mentioned in Supplementary Fig. S4a. The attachment process of the Brainmask device ( ~ 1.35 mm in thickness) to the wave board is shown in Supplementary Movie 4, with conformal contact results from both side view and top view photos in Fig. 4c. The transparent model of the deformed device attached to the wave board on top view is illustrated in Fig. 4d, as well as the other side of the device with electrodes on top of the BC film with the aid of a thin layer of soft adhesive Ecoflex Gel (Fig. 4e). Owing to the thin soft adhesive layer, the independent electrode channels can still maintain accurate positions along with a large curvature deformation. As shown in Fig. 4f and Supplementary Movie 5, the strain of the Au layer in the valley area is larger than the peak but far from plastic deformation, even for the interconnects close to the electrode pads. The side view of the global z-axis displacement of the device in Fig. 4g further illustrates its conformality to the wave board. Moreover, the von Mises stress distribution of the Ecoflex Gel and BC layers is exhibited in Fig. 4h, with peak values of 45 kPa and 26 kPa, respectively. This proves the advantage of the ultrasoft dual-layer substrate to fit any curved surface as well as the minimal effect it has on the accurate positioning of serpentine electrodes.

### Morphology characterization of the BC film

**Fig. 5:**
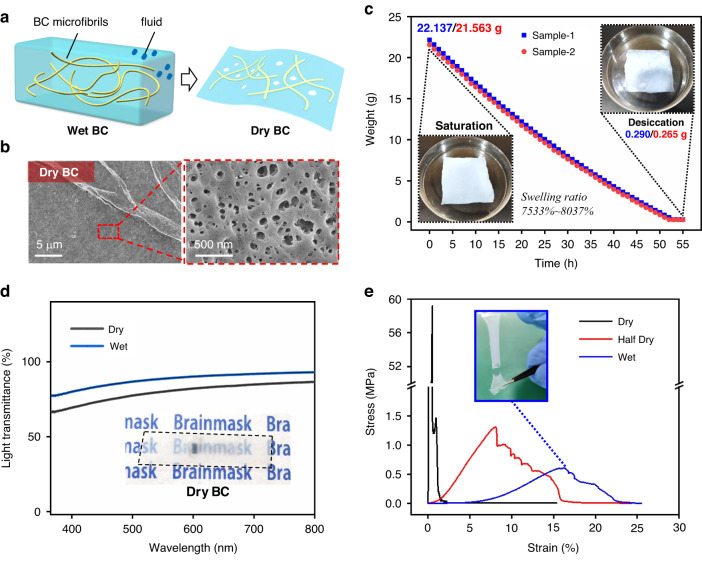
Morphology, moisture retention, transparency and tensile strength of the BC film as the substrate of the Brainmask device. **a** Schematic diagram of the internal structure of the BC film in wet and dry states. **b** Dry BC film characterized by SEM with distributed nanopores. **c** The weight of the BC film from the saturation to desiccation state with time. **d** Transmittance of visible light (wavelength from 390 nm to 780 nm) through the dry and wet BC films characterized by spectrophotometer. **e** Stress‒strain curves of the dry, half-wet and wet BC films measured by the in situ tensile test, with an inset photo of the fractured wet sample

A BC film is the product of fermentation and culture of *Gluconobacter*. As illustrated in Fig. 5a, the schematic diagram of the internal structure of the wet BC film shows that the BC microfibrils are uniformly dispersed in the fluid after swelling, and its own weight can reach over 100 times that of the dry state. Meanwhile, the initial thickness of the swelling BC film that we used reached 2.86 mm, while the thickness of the dry film decreased to 30 *μ*m after natural-draft drying or heating. Furthermore, the dry BC film was characterized by scanning electron microscopy (SEM) with equally distributed nanopores, which are formed by the overlapping BC microfibrils, as pictured in Fig. 5b. These subtle nanopores are beneficial for the infiltration of silicone gel and increase the bonding force at this interface to avoid delamination.

### Moisture retention of the BC film

The moisture retention of BC film is a unique feature compared to other substrate materials. When the BC film is immersed in water, it can maintain saturation alone. However, the moisture of the wet BC film still slowly evaporates with its weight and thickness reduced in the air. To explore the water loss rate of the BC film with time, we placed two saturated samples 8 × 8 cm^2^ in size at 25 °C in air without direct sunlight and measured the weight on an electronic balance every hour. The measured weights of the two BC samples from the saturation to desiccation state are plotted in Fig. 5c. The initial weights of the two samples are 22.137 g and 21.563 g at the saturation state, but they stabilize at 0.290 g and 0.265 g after more than 50 h, respectively, with swelling ratios of 7533%-8037%. It is clear that the weight loss is close to linear with time, and the loss rate is slow enough for acute implantation with exposed brain tissue in air. In practice, the wet BC substrate in the Brainmask device can greatly reduce the evaporation of cerebrospinal fluid (CSF), which is vital for the protection of neurons and the immune microenvironment of the brain.

We notice that regardless of the ratio of water loss during evaporation, as long as water remains in the BC film, it can always recover to the initial thickness similar to the saturation state. However, the water absorption capacity of the completely dried BC film will decline, and it can hardly recover to the initial thickness. Because it is necessary to print silicone gel and transfer the serpentine electrode onto the dry BC film, we evaluated the thickness of the BC film in a water absorption test at different temperatures. The samples were immersed in deionized water at temperatures of 20, 40, 60, 80 and 100 °C with the aid of a stir bar at a fixed speed of 400 r/min for 30 min. As pictured in Supplementary Fig. S8a, the water absorption effect of the dry BC film is obviously improved with increasing water temperature. The thickness values of BC with water temperatures from 20 °C to 100 °C are plotted in Supplementary Fig. S8b. When the temperature is above 60 °C, the increase in thickness gradually slows down and reaches a steady value of approximately 1.4 mm, which is half of the wet BC. Thus, we chose 80 °C hot water for reabsorption and obtained the final wet BC substrate for the Brainmask device with a thickness of 1442.75 ± 58.26 *μ*m, as listed in Supplementary Table S1.

### Transparence of BC film

It is necessary to ensure the transparency of the Brainmask device when implanting it in the target area. We used ultraviolet spectrophotometry to detect the light transmittance of the BC film in both dry and wet states. The transmittance of visible light (wavelength from 390 nm to 780 nm) through the dry and wet BC films was characterized by spectrophotometer for comparison in Fig. 5d. Taking a wavelength of 560 nm (for standard measurement of plate glass) as an example, the transmittances of dry and wet BC films are 80.5% and 89.0%, respectively. This demonstrates the advantage of the high transparency of the BC film, which is even better in the wet state for clear observation. As seen from the inset photo, the serpentine electrode as well as the text “Brainmask” can be distinguished beneath the dry BC film. It is conducive to targeting the implantation location during the operation and improving the spatial accuracy.

### Tensile strength of BC film

The Brainmask device will be placed beneath the skull and attached to the surface of the cerebral cortex; thus, we have to consider the stretching caused by the micromotion of brain tissue, as well as the stretching caused by the connection area with the FPC encapsulated by the solidified dental cement. Therefore, the tensile strength of the BC film as a substrate needs testing to ensure that it is not vulnerable to damage or even failure after implantation. It is noted that the mechanical properties of the BC film vary with different moisture ratios, so we tested the samples under dry (desiccation, moisture 0%), half dry (24 h in air from saturation, moisture ~50%) and wet (saturation, moisture 100%) states. The stress‒strain curves of these three samples are plotted in Fig. 5e, with an inset photo of the fractured wet sample. The dry BC film exhibits brittle failure with an instantaneous peak stress close to 60 MPa, but it is almost unstretchable. As the moisture ratio increases, the BC film becomes softer and more stretchable, as large as 8% for the half dry sample and 16% for the wet sample, but the peak stress declines to approximately 0.5 MPa for the wet sample. However, it is strong enough to withstand the minute force that occurred during and after implantation. To further prove mechanical stability, the wet Brainmask sample (length of 30 mm, width of 20 mm) was fixed for cyclic tensile testing at a 10% strain (Supplementary Fig. S9), and the measured tensile force was stabilized at 0.143 N (Supplementary Fig. S10) without any further degradation. Therefore, the wet BC film is an ideal candidate that combines the excellent properties of ultrasoftness, moisture retention, transparency and tensile strength to meet the requirements of long-lasting operations in vivo.

### Electrochemical properties

**Fig. 6:**
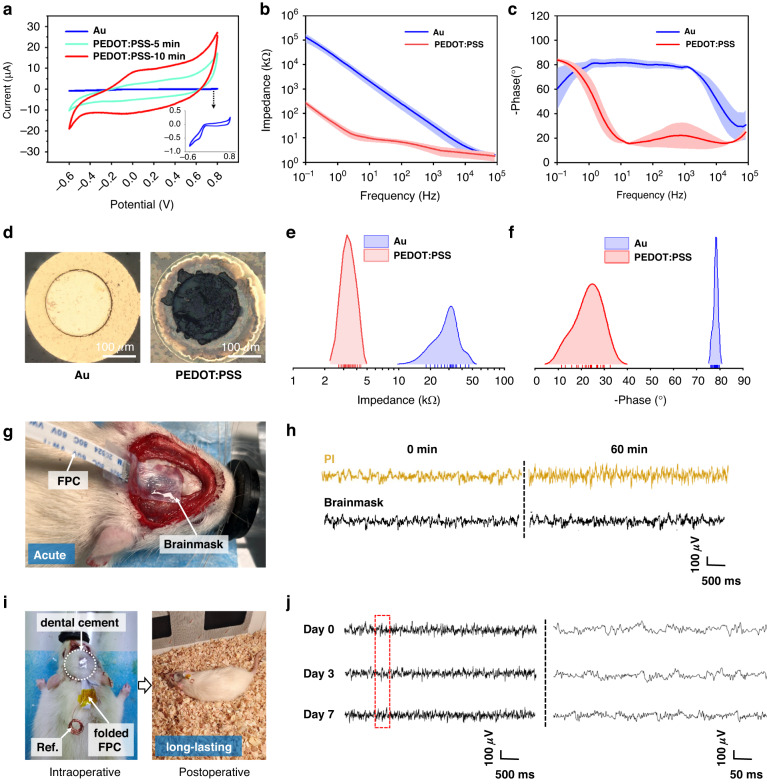
Electrochemical test and in vivo implantation of the Brainmask device. **a** Cyclic voltammetry curves of bare gold and electrochemical deposition of PEDOT:PSS for 5 and 10 min with a constant deposition current density of 0.4 mA·cm^−2^. Comparison of (**b**) impedance and (**c**) phase curves of the bare gold and PEDOT:PSS (10 min) with an error band (*n* = 45). **d** Photos of the microelectrode site before and after PEDOT:PSS modification. Distribution of (**e**) impedances and (**f**) phases for bare gold and PEDOT:PSS (*n* = 45) measured at 1 kHz. **g** Photo of the acute implantation of the Brainmask device on the epidural surface of a rat under anesthesia. **h** Comparison of the acute ECoG signals acquired from the PI-based device and BC-based Brainmask device before and after the cranial window was exposed to air for 60 min. **i** Photos of the Brainmask device with folded FPC left outside for long-lasting recording in vivo. **j** Comparison of the ECoG signals acquired on Day 0, Day 3 and Day 7

To reduce the impedance and improve the signal-to-noise ratio (SNR), the bare gold microelectrodes were electrochemically modified by PEDOT:PSS with a constant deposition current density of 0.4 mA/cm^2^ for 5 min and 10 min. After electrodeposition, the cyclic voltammetry (CV) curves, as plotted in Fig. 6a, indicate a much larger encircled area for PEDOT:PSS than for bare gold. The charge storage capacity (CSC) values are calculated as 11.69 mC/cm^2^ and 26.04 mC/cm^2^ for modification times of 5 min and 10 min, respectively, which improve significantly compared with 0.71 mC/cm^2^ for bare gold. In addition, the stability of the PEDOT:PSS coating was verified by synchronous CV scanning between −0.6 V and 0.8 V in PBS solution with a scanning rate of 1 V/s, as well as in an ultrasonic bath (100 W, 40 kHz). The CV curves before and after 200, 400 and 600 CV scanning cycles are compared in Supplementary Fig. S11. The curves tend to be stable with increasing scanning cycles, and no obvious cracking, delamination or exfoliation phenomena occur on the PEDOT:PSS coating. In addition, electrochemical impedance spectroscopy (EIS) was used to compare the impedance characteristics of bare gold and PEDOT:PSS (10 min); 5 samples with 45 microelectrode sites were tested in total. The impedance and phase curves are plotted in Fig. 6b, c, and the distribution statistics of impedances and phases at 1 kHz are shown in Fig. 6e, f, respectively. Specifically, the impedances are 30.8 ± 7.0 kΩ and 3.5 ± 0.5 kΩ, and the phases are −78.2 ± 0.9° and −23.2 ± 5.8° for Au and PEDOT:PSS at 1 kHz. In addition, the photos of the microelectrode site before and after modification are compared in Fig. 6d. Thus, the modification is effective and reliable for long-lasting recording.

### In vivo ECoG recording

To validate the Brainmask device, acute (1 h) and long-lasting (1 week) recordings in rats were conducted. Supplementary Movie 6 shows the process of easy conformal attachment of the wet Brainmask device on the epidural surface of the rat cortex under anesthesia, with the result pictured in Fig. 6g. To illustrate the advantage of the ultrasoft and moist BC-based Brainmask device, a pure PI-based device with the same microelectrodes was used for comparison. Micro-ECoG signals were recorded at a sampling rate of 1 kHz and filtered to remove power frequency interference. Meanwhile, the data were processed via the EEGLAB package with a frequency pass band of 0.5–200 Hz. The recording area is the somatosensory cortex, and the corresponding signals of the 4-channel Brainmask device are illustrated in Supplementary Fig. S12. The acute signals were acquired before and after the cranial window was exposed to air for 60 min, as shown in Fig. 6h. The SNR value of the PI-based device decreased from 2.91 ± 0.05 dB at 0 min to 2.48 ± 0.05 dB at 60 min. In comparison, the SNR value of the Brainmask device decreased from only 3.14 ± 0.06 dB at 0 min to 3.06 ± 0.06 dB at 60 min, with the quantification method described in the Supplementary information. It is clear that the signal remains satisfactory with a higher SNR for Brainmask after exposure in air, owing to the moisture retention at the contact interface, which shows its unique advantage during long-lasting recording. Then, the Brainmask device was fixed and sealed with a soft PDMS patch and dental cement in sequence, leaving a folded FPC outside for follow-up recording postoperatively, as pictured in Fig. 6i. The signals were recorded on Day 0, Day 3 and Day 7, as compared in Fig. 6j. To our satisfaction, the signal remained stable on Day 7, and the rat still lived without any unusual signs. This preliminarily verifies the long-lasting stability of the Brainmask device to record cortical ECoG for both acute and chronic occasions.

## Discussion

In this work, we propose an innovative minimally invasive BCI device named Brainmask, with the advantages of accurate positioning of thin-film serpentine electrodes on a BC film and long-lasting micro-ECoG recordings. The ultrasoft wet BC film as a substrate can fit well on a nondevelopable surface, e.g., a ball. By using both experiments and simulations, we further verified the flexible device by twisting 180° and attaching it onto a wave board with satisfactory deformable properties. The BC film demonstrated excellent moisture retention to avoid a decline in signal quality, high transparency for targeting the implantation location during the operation, and a strong tensile strength of 0.5 MPa and stretchability of 16% for the wet BC film. Finally, the Brainmask device was modified with PEDOT:PSS for high SNR and implanted in rats for long-lasting recordings from hours to days; satisfactory signals were exhibited in both cases. Therefore, we believe that this novel Brainmask device is promising for replacing common polyimide or parylene electrodes. It has addressed two present critical issues on conformality and moisture for micro-electrocorticography recordings.

## Materials and Methods

### Preparation of the bacterial cellulose (BC) film

The wet BC membrane was purchased from Wenchang Baocheng Ltd. (China). The membrane was stored in a low-temperature environment at 4 °C and sealed in a Ziplock bag to avoid water evaporation and bacterial pollution. At the beginning of the experiment, the BC membrane was dehydrated at room temperature or in an oven and then completely dried into an approximately transparent sheet film after a period of time. The film was cut into strips 12 mm in width and 15 mm in length for the subsequent assembly.

### Water loss test of BC

An electronic analytical balance (FA-N, Lichen Ltd., China) was used to test the water loss rate of the BC membrane at room temperature. The BC membrane was cut into 8 cm × 8 cm squares, two pieces in total. The samples were placed in a beaker to fully absorb water, laid in two glass dishes to evaporate naturally at room temperature of 25 °C and ambient humidity of 50% rh, and weighed every hour from the saturation to desiccation state.

### Water absorption test of BC

To explore the water absorption capacity of the BC membrane after dehydration, the completely dried BC membrane was cut into strips, placed in deionized water at 20 °C, 40 °C, 60 °C, 80 °C and 100 °C, and stirred by magnetic stirring in an oil bath pot. Then, the strip samples were measured with respect to thickness by calipers to evaluate the degree of water absorption.

### Light transmittance test of BC

The absorbance of the BC film was measured at wavelengths from 390 nm to 780 nm by a spectrophotometer (UV2450, Shimadzu, Japan). Light transmittance curves of the dry and wet BC films were plotted for comparison.

### Tensile test of BC

First, the micro-in multiscale mechanical testing system (IBTC-300SL, Care Measurement & Control Co., Ltd., China) was used to test the tensile strength of dry, half-dry and wet BC film samples, with two-end clamps moved symmetrically to the opposite side and the sample always located in the center of the field of view. All samples were prepared with a length of 30 mm and width of 15 mm. The wet BC film was measured in the saturation state, the half-dry BC film was placed at a room temperature of 25 °C and an ambient humidity of 50% rh for 24 h, and the dry BC film was in the desiccation state. The stress‒strain curves were plotted until the samples stretched to the breakage point. In addition, cyclic tensile testing of the wet Brainmask at 10% strain was also conducted on this mechanical testing system.

### Fabrication of the serpentine electrode

The microelectrode array was fabricated with the MEMS flows of deposition and etching of polymer and metal thin films (as shown in Supplementary Fig. S1). First, 500 nm thick aluminum was evaporated on a four-inch silicon wafer by physical vapor deposition as the sacrificial layer, followed by the chemical vapor deposition (CVD) of 5 *μ*m thick parylene-C as the insulating substrate layer. To improve the bonding force between parylene-C and the metal layers, the wafer was treated with O_2_ plasma (40 W, 1 min) to improve the surface roughness of the parylene-C film.

Then, Cr/Au (30/200 nm) was deposited by metal magnetron sputtering. The 4-channel and 9-channel electrodes were designed on the same mask, with a serpentine interconnect layout to ensure deformability. The metal layer was covered with patterned positive photoresist (3 *μ*m) by photolithography and dry etched by an ion-milling system (IBE-150B, AdvancedMEMS, China) to form the wire interconnection.

Next, O_2_ plasma treatment (40 W, 1 min) was performed again, followed by CVD of the second layer of 3 *μ*m thick parylene-C as the encapsulation layer. Another layer of positive photoresist (8 *μ*m) was used as a mask to pattern both parylene-C layers by reactive ion etching (RIE, 100 W, 15 min). The serpentine interconnects were shaped with metal microelectrode sites and pads exposed.

Finally, acetone was used to remove the residual photoresist, followed by the sacrifice of the aluminum layer in 5% hydrochloric (HCl) acid.

Simultaneously, the same serpentine layout was adopted to fabricate the microelectrode array based on the polyimide (PI) substrate. The fabrication flows only differed in the pattern method of the two sandwiched PI layers, using photosensitive PI (HD 4100, HD MicroSystem, Japan) instead of RIE-patterned parylene-C. After spin-coating (3800 r/min, 30 s), prebaking (90 °C, 100 s and 100 °C, 100 s), photolithography, development (YS-108, Suzhou Yilan Microelectronics Co., Ltd, China) and rinsing (YS-923, Suzhou Yilan Microelectronics Co., Ltd., China), the patterned bottom PI layer was incompletely cured in N_2_ at 300 °C for 60 min to an approximately 5 *μ*m in thickness. For the top PI encapsulation layer, the same flow was applied with a final cure temperature of 350 °C for 60 min. The total thickness of the PI-based electrodes was 10 *μ*m, slightly thicker than that of 8 *μ*m for the parylene-based electrodes.

### Thickness measurements and SEM morphology

The three Brainmask samples were measured in terms of thickness with a surface profiler (Dektak XT, Bruker, USA), including the wet and dry BC substrates, the adhesive gel layer, the sandwiched Parylene-C encapsulations, and the polyimide encapsulations, as listed in Supplementary Table S1. The surface morphology of the dry BC film was imaged by SEM (VEGA 3 LMU, Teskan, Czech Republic).

### Conformality on a nonplanar surface

In the first experiment, the adhesion effect was measured for PDMS (*E* = 1.0 MPa), Ecoflex (*E* = 0.06 MPa) and wet BC (*E* = 0.08 MPa) samples with the same thickness of 1.4 mm and width of 12 mm on the developable surface of a cylinder (diameter of 10 mm) and the nondevelopable surface of a ball (diameter of 20 mm). In the second experiment, the adhesion effect was measured for Ecoflex and wet BC samples with the same thickness of 1.4 mm on the large (2.5 mm in radius) and small (0.7 mm in radius) wave boards, respectively, to further prove the conformal advantage of a wet BC film on a nonplanar surface, even smaller than the radius of curvature of the rat brain.

### Mechanical finite-element analysis

ABAQUS commercial software (ABAQUS Analysis User’s Manual 2010; V6.16) was used to study the mechanical characteristics of the assembled Brainmask device. Ogden’s hyperelastic model was used to model the mechanical response and was easily applied to accurately model the stress–strain response of the BC film, considering the hyperelastic constants of the constitutive model for BC. Meanwhile, no restriction was imposed on the material constants, and the Drucker stability criteria, available in ABAQUS, were used to ensure model stability. The Mooney-Rivlin model was used to represent the hyperelastic soft adhesive layer of Ecoflex Gel with the following parameters: *C*_*10*_ = *E*/5(1+*ν*), *C*_*01*_ = *E*/20(1+*ν*) and *D*_*1*_ = 6(1-2*ν*)/*E*. Linear elasticity was used to define the parylene-C film, and the elastic‒plastic model was used to model the Au metal layer. On the one hand, the device was twisted 180° to demonstrate the influence of large deformation on the strain distribution of the Au layer. On the other hand, the device was attached to a wave board surface with a radius of 2.5 mm to validate the conformality and mimic the sulcus gyrus of the rat cortex.

### Electrochemical characterization

The electrochemical experiments of the electrodes were conducted on an electrochemical workstation (CHI600E, CH Instruments, China) with a conventional three-electrode setup; the Brainmask device was the working electrode, the Pt sheet was the counter electrode and the saturated calomel electrode (SCE) was the reference electrode in phosphate-buffered saline (pH 7.2–7.4) at room temperature. Cyclic voltammetry (CV) was scanned over the potential range of −0.6 V and 0.8 V at a scanning rate of 100 mV/s. Electrochemical impedance spectroscopy (EIS) experiments were performed in the frequency range of 0.1 Hz–100 kHz with an AC excitation voltage amplitude of 10 mV at the open circuit potential (OCP), presented in the form of impedance and phase.

### Electrodeposition of PEDOT:PSS

Five Brainmask samples with 45 microelectrode sites in total were used for modification and measurement. All sites were electrodeposited with poly(3,4-ethylenedioxythiophene) poly(styrenesulfonate), namely, PEDOT:PSS, as a coating to reduce the impedance and improve the SNR. First, 5 mg/ml polystyrene sulfonate (PSS) powder was added to 50 ml deionized water and stirred to dissolve. Then, 0.01 M EDOT was added, and the solution was uniformly mixed for 2 h using a stirring bar to obtain an electrolyte. Prior to electrodeposition, 50 CV cycles were performed simultaneously on all channels from −1.0 V to 1.0 V versus SCE with a 1 V/s scanning rate in phosphate buffered saline (PBS, pH 7.4) as a cleaning step. The PEDOT:PSS composite was electrodeposited by the galvanostatic method in a three-electrode cell with the Brainmask sample as the working electrode, Pt mesh as a counter electrode and SCE as a reference electrode. The deposition was carried out on an electrochemical workstation by applying a constant current with a deposition current density of 0.4 mA/cm^2^ for at most 10 min.

### In vivo animal experiments

Two male Sprague‒Dawley (SD) rats weighing 250 g were used in the experiments. All experimental procedures were approved by and performed in accordance with the guidelines established by the Medical and Experimental Animal Ethics Committee, Northwestern Polytechnical University, Shaanxi, China. The rat was anesthetized with isophane gas and fixed on the experimental platform, and oxygen mixed with isophane gas was continuously infused into its nasal cavity. The right parietal bone was removed by craniotomy with a surgical drill, and a cranial window of approximately 4 × 4 mm^2^ was created with the dura mater preserved. The skull screw was fixed at the posterior end of the parietal bone with a copper wire for grounding. The Brainmask device was placed on the cranial window, followed by covering a soft PDMS patch on top to avoid direct contact of the hardened dental cement with the moist BC substrate. Then, mixed dental cement (Peolangkg, Changshushangchi Dental Materials Co., Ltd., China) was applied to solidify and seal the exposed cranial window with the folded flexible printed cable (FPC) left outside and stacked on top of the rat’s head. After surgery, the rat was put into a shield cage to shield the external electromagnetic interference, especially the 50 Hz power interference, for the convenience of high SNR signal recording. The micro-ECoG signals were acquired while freely behaving for one week, on Day 0, Day 3 and Day 7, to preliminarily verify the reliability of chronic implantation.

### Supplementary information

supplementary information

Video 1 Serpentine electrode without BC attaching on the cylindrical surface of agar gel

Video2 Serpentine electrode with BC attaching on the cylindrical surface of agar gel

Video3 Simulation result of the Brainmask when twisted 180 degrees

Video4 Experiment result of the Brainmask attaching on the wave board

Video5 Simulation result of the Brainmask attaching on the wave board

Video6 Brainmask easily attached on the epidural surface of rat cortex

## Data Availability

The data that support the findings of this study are available from the corresponding authors upon reasonable request.
