# Supplementary material for: Brainmask: an ultrasoft and moist micro-electrocorticography electrode for accurate positioning and long-lasting recordings
Source: Microsyst Nanoeng. 2023 Oct 10;9:126. doi: 10.1038/s41378-023-00597-x (PMC10564857; doi:10.1038/s41378-023-00597-x)
Supplement: Supplementary file 1 — supplementary information [file 41378_2023_597_MOESM1_ESM.docx]

**Supplementary information**

Brainmask: an ultrasoft and moist micro-electrocorticography electrode for accurate positioning and long-lasting recordings

Bowen Ji^1, 2, 3, #^, Fanqi Sun^1, 2, 3, #^, Jiecheng Guo^4, #^, Yuhao Zhou^1, 2, 3^, Xiaoli You^1, 2, 3^, Ye Fan^5^, Longchun Wang^6^, Mengfei Xu^6^, Wen Zeng^2^, Jingquan Liu^6^, Minghao Wang^5, *^, Huijing Hu^4, *^, Honglong Chang^2, *^

^1^ Unmanned System Research Institute, Northwestern Polytechnical University, Xi’an 710072, China

^2^ Ministry of Education Key Laboratory of Micro and Nano Systems for Aerospace, School of Mechanical Engineering, Northwestern Polytechnical University, Xi’an 710072, China

^3^ Collaborative Innovation Center of Northwestern Polytechnical University, Shanghai 201108, China

^4^ Institute of Medical Research, Northwestern Polytechnical University, Xi’an 710072, China

^5^ College of Electronics and Information, Hangzhou Dianzi University, Hangzhou 310018, China

^6^ National Key Laboratory of Science and Technology on Micro/Nano Fabrication, Department of Micro/Nano Electronics, Shanghai Jiao Tong University, Shanghai 200240, China

^#^ B. J., F. S. and J. G. contributed equally to this work.

^*^Correspondence: mhwang@hdu.edu.cn (M. Wang); huhuijing@nwpu.edu.cn (H. Hu); changhl@nwpu.edu.cn (H. Chang)

**Table S1** Parameters of the material thicknesses in the Brainmask device.

| **Materials** | **Thickness (*μ*m)** |
| --- | --- |
| Wet BC (reabsorption in 80℃ hot water) | 1442.75±58.26 |
| Dry BC | 27.91±3.19 |
| Ecoflex Gel (adhesive layer) | 153.16±19.58 |
| Parylene-C (bottom 5 *μ*m+top 3 *μ*m) | 7.89±0.15 |
| Polyimide (bottom 5 *μ*m+top 5 *μ*m) | 9.94±0.22 |

**Calculation of the SNR**

The SNR can be extracted from the power spectral density (PSD). The PSD was estimated using pwelch method (${\mu V}^{2}\mathrm{Hz}^{-1}$). For the frequency band in [$f_{1}, f_{2}$]

$$P_{[f_{1}, f_{2}]}=\left( \int_{f_{1}}^{f_{2}} dfS\left( f \right) \right)/ (f_{2}-f_{1})$$

where *S(f)* is the power spectrum, and then $P_{[f_{1}, f_{2}]}$ is presented in 10 log10 scale:

$$P_{[f_{1}, f_{2}]}=10{log}_{10}\left( P_{\left[ f_{1}, f_{2} \right]} \right)[dB]$$

The SNR ratio was calculated as the ratio of the signal $P_{signal}$ versus the noise $P_{noise}$ in dB:

$$SNR=10{log}_{10}\frac{P_{signal}}{P_{noise}}$$

We estimated the signal band $P_{signal}$ in the 0.5-200 Hz frequency band. The noise band $P_{noise}$ in the 250-260 Hz frequency band was estimated because of the digital filter of the Integrated Circuit (cut-off frequency 300 Hz) which starts altering the signal at above ~250-260Hz ^[s1-s2]^.


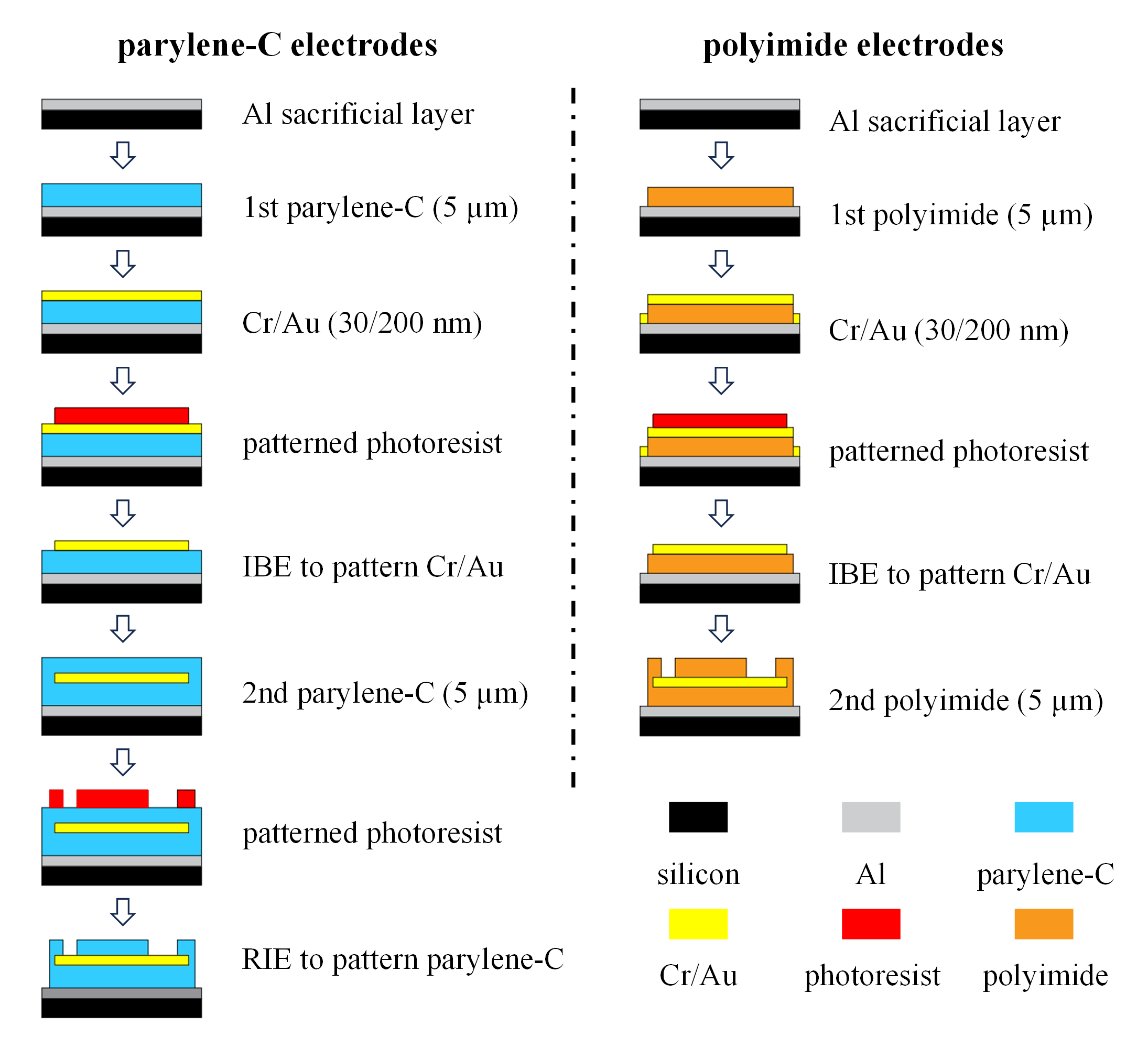


**Fig. S1** MEMS fabrication processes of the serpentine parylene-C and polyimide electrodes.


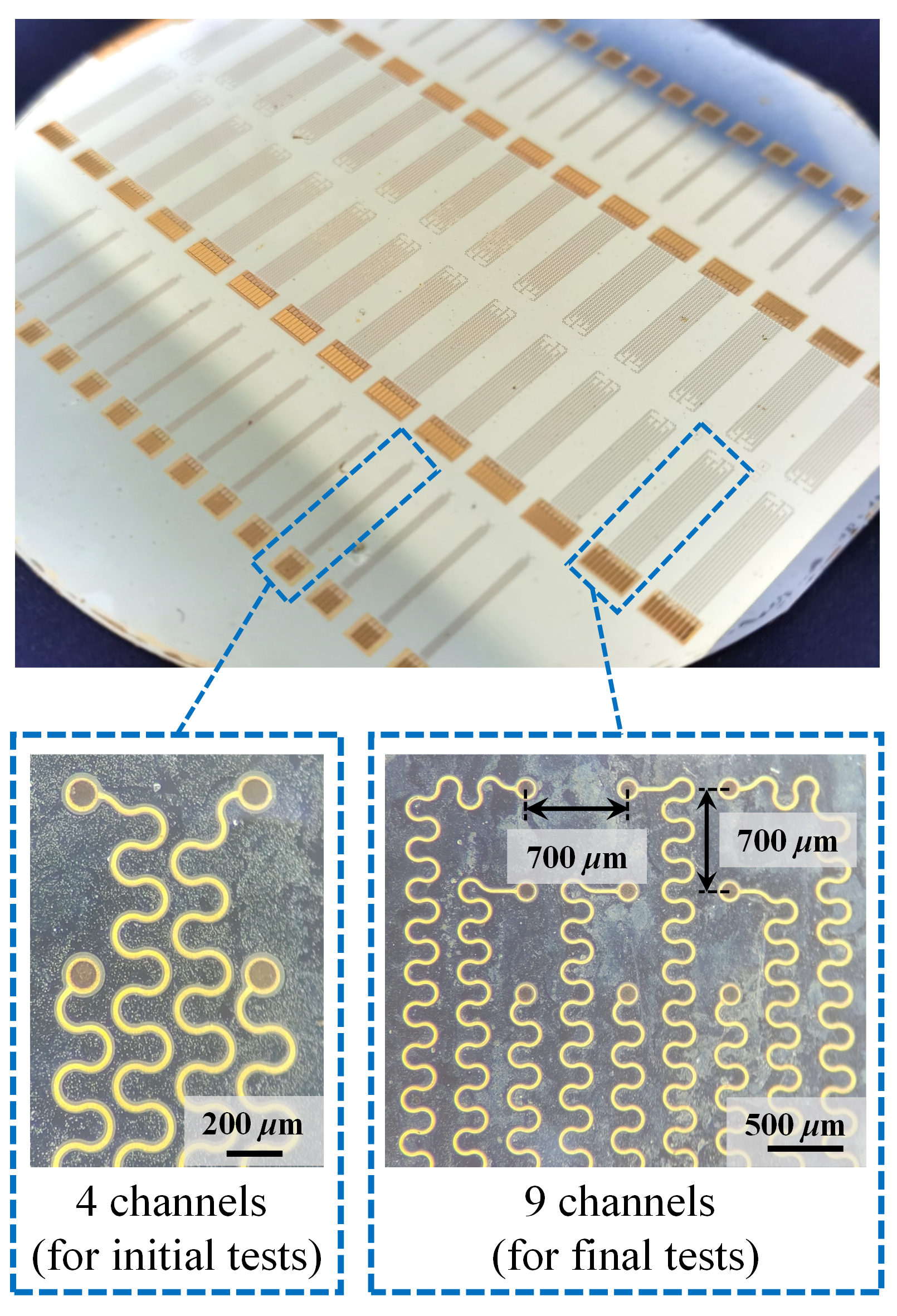


**Fig. S2** The MEMS-fabricated stretchable micro-ECoG electrodes based on parylene-C thin film, with thickness of 8 *µ*m.


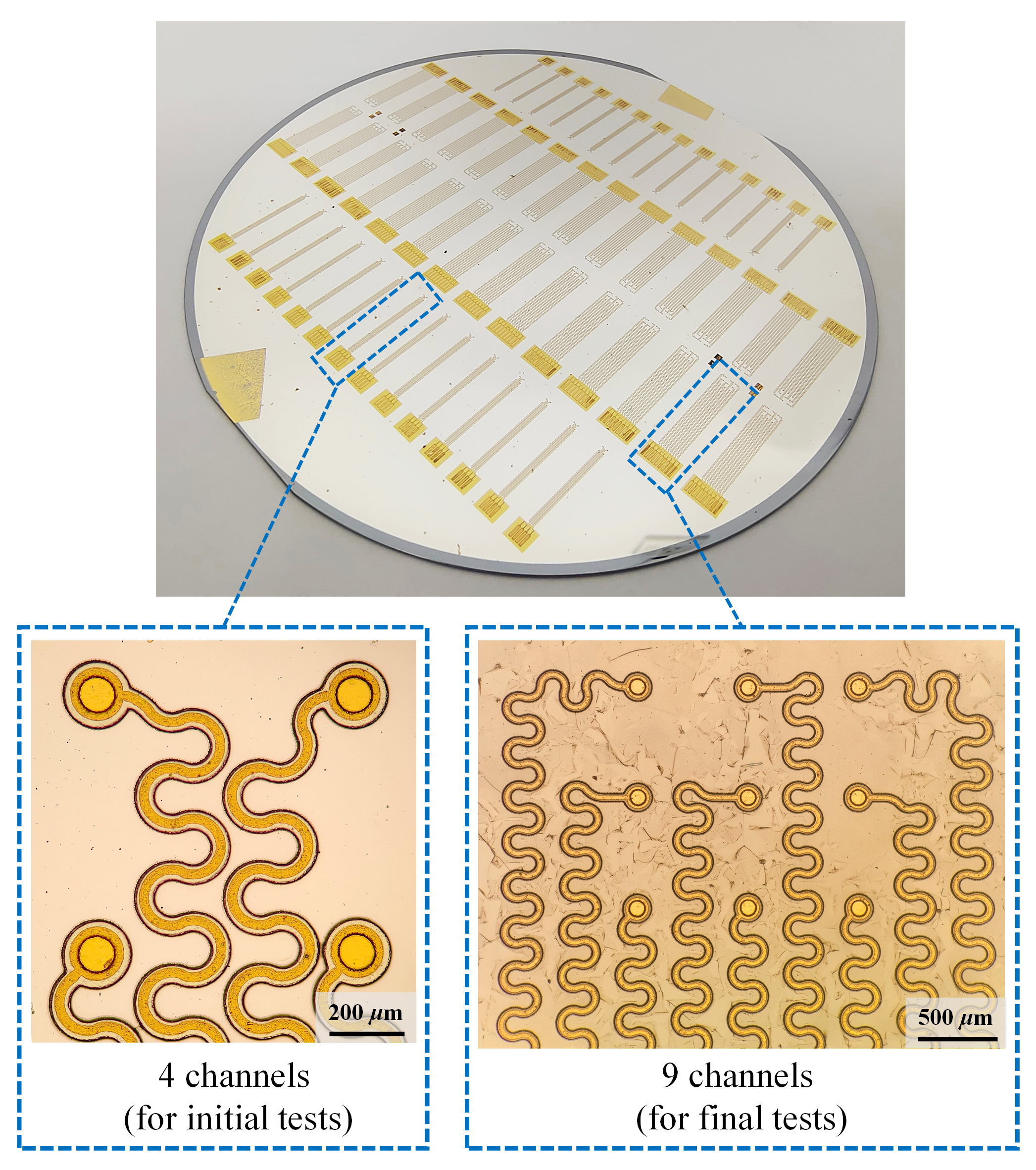


**Fig. S3** The MEMS-fabricated stretchable micro-ECoG electrodes based on polyimide thin film, with thickness of 10 *µ*m.





**Fig. S4** Relative position of the Ecoflex gel under the front edge region and the conductive electrode pads in the pad region.


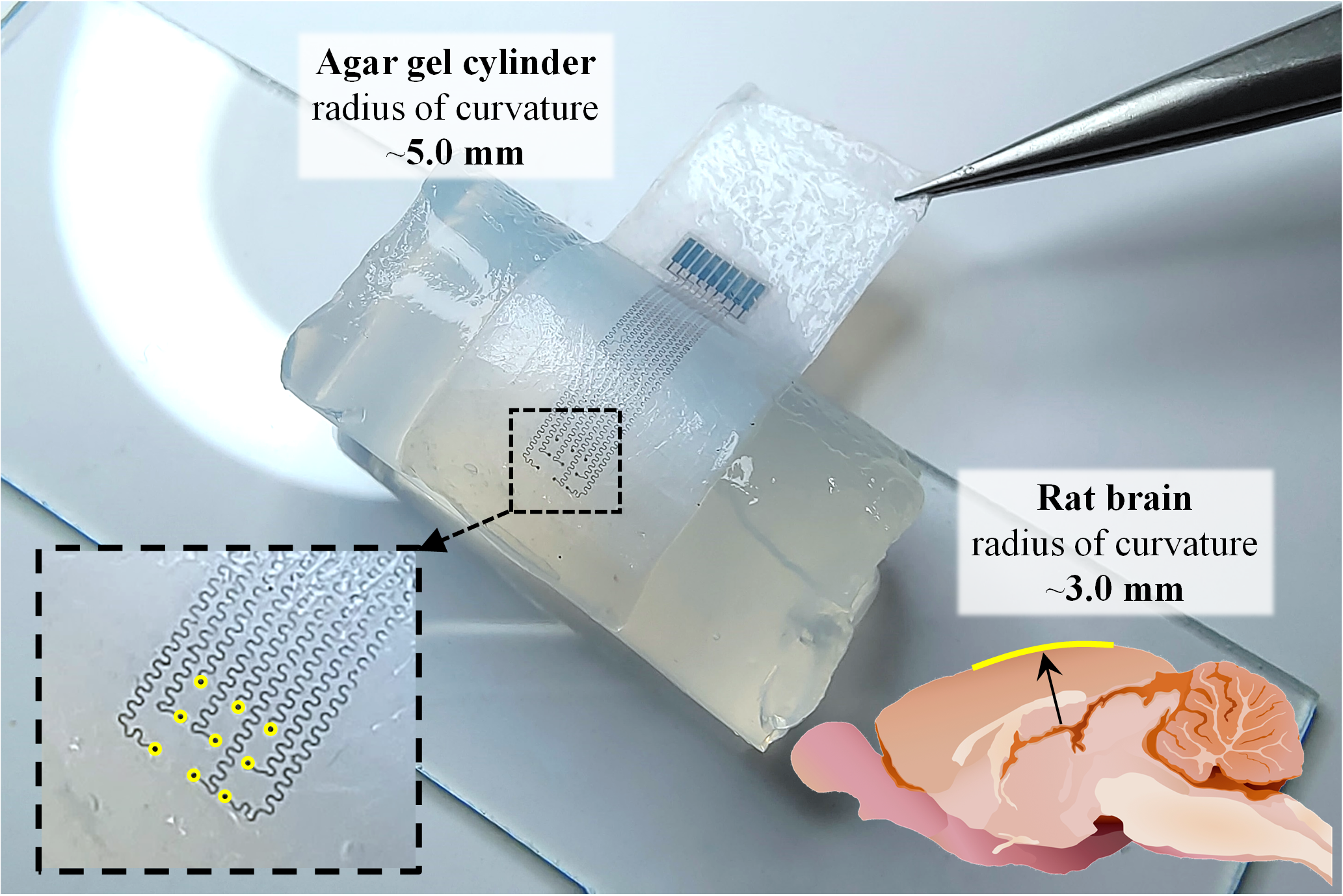


**Fig. S5** Conformal attachment of the Brainmask to the agar gel cylinder (mimic brain tissue) with similar radius of curvature to the rat brain.


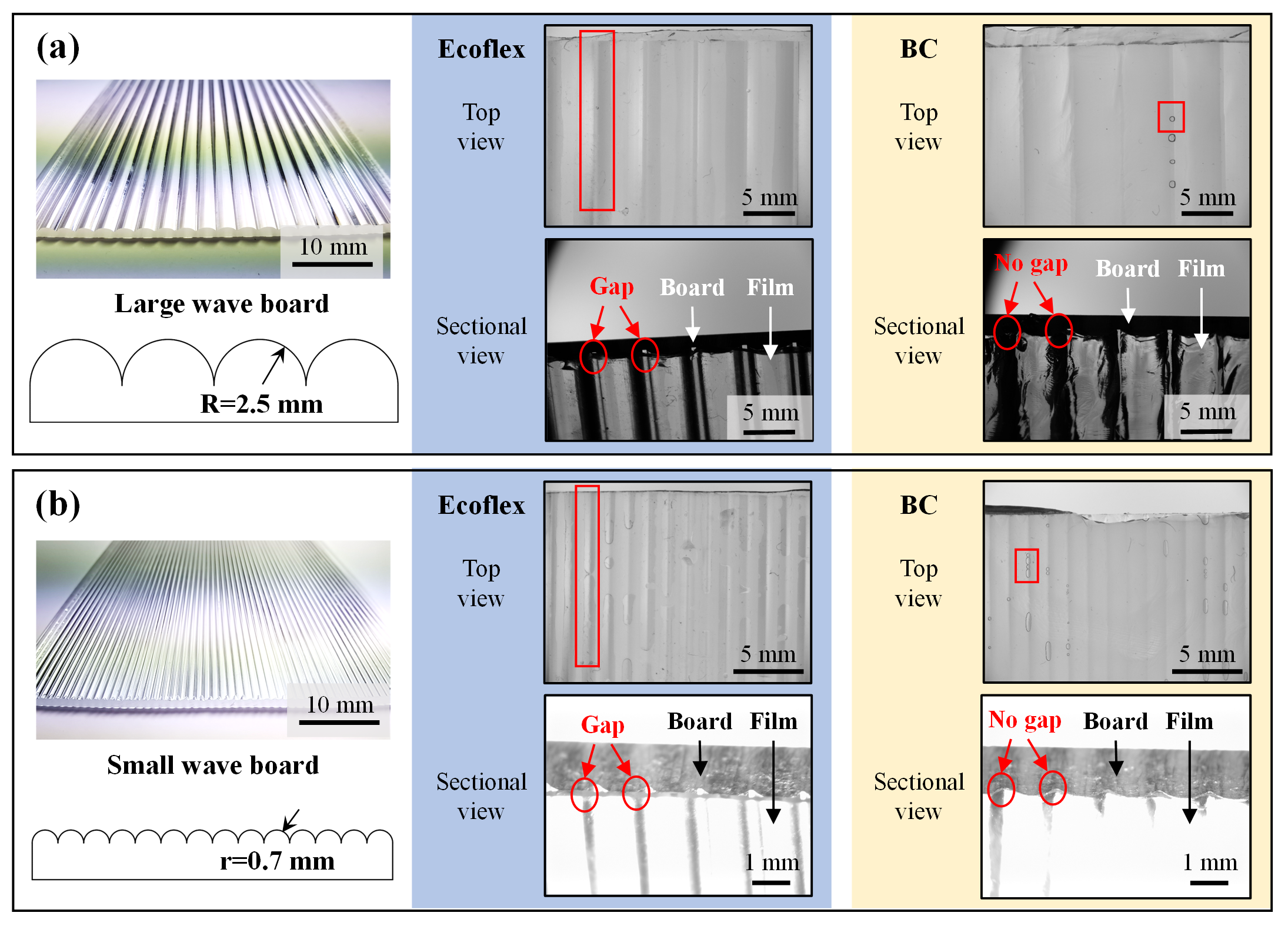


**Fig. S6** Comparison of Ecoflex and wet BC films adhered to the (**a**) large (2.5 mm in radius) and (**b**) small (0.7 mm in radius) wave boards from top and sectional views. Both Ecoflex and BC films are about 1.4 mm in thickness.


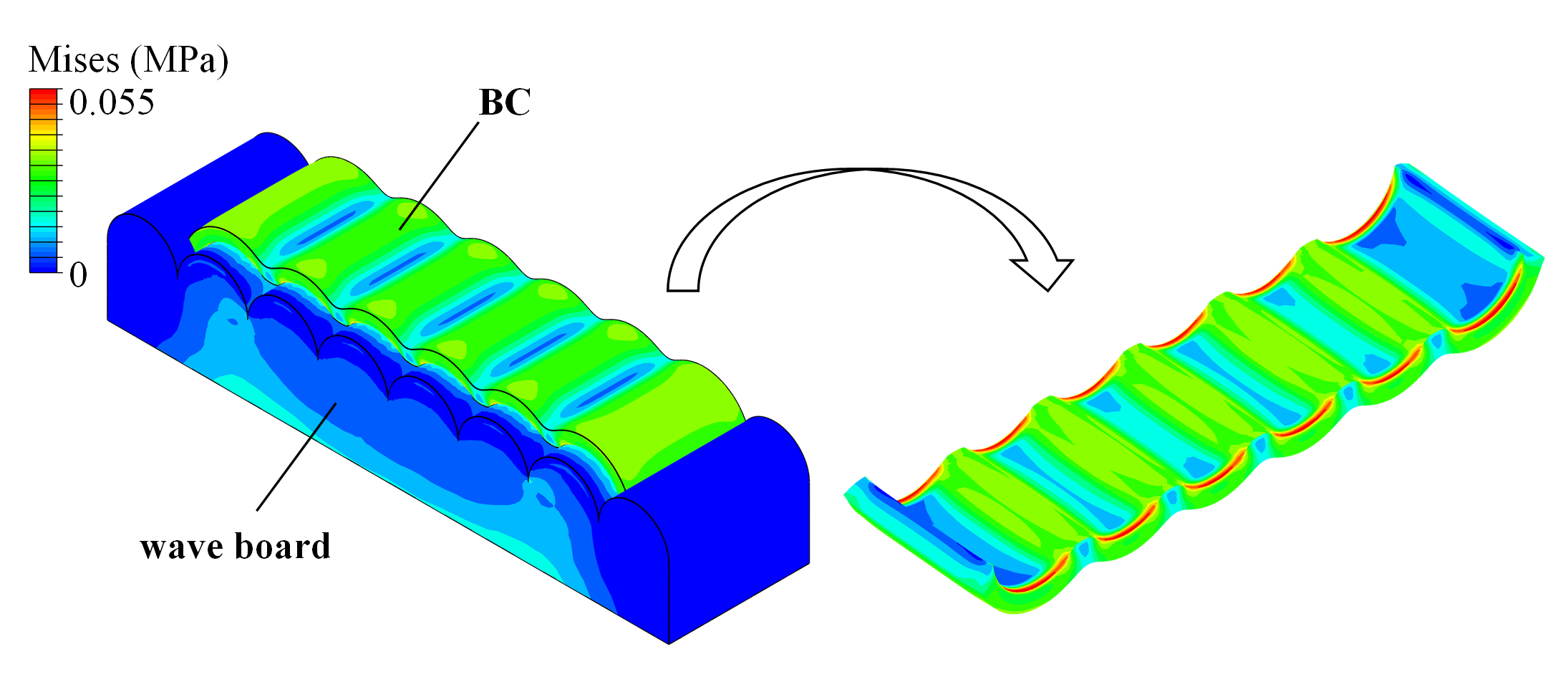


**Fig. S7** Mises stress of the BC film adhered to the wave board (2.5 mm in radius).


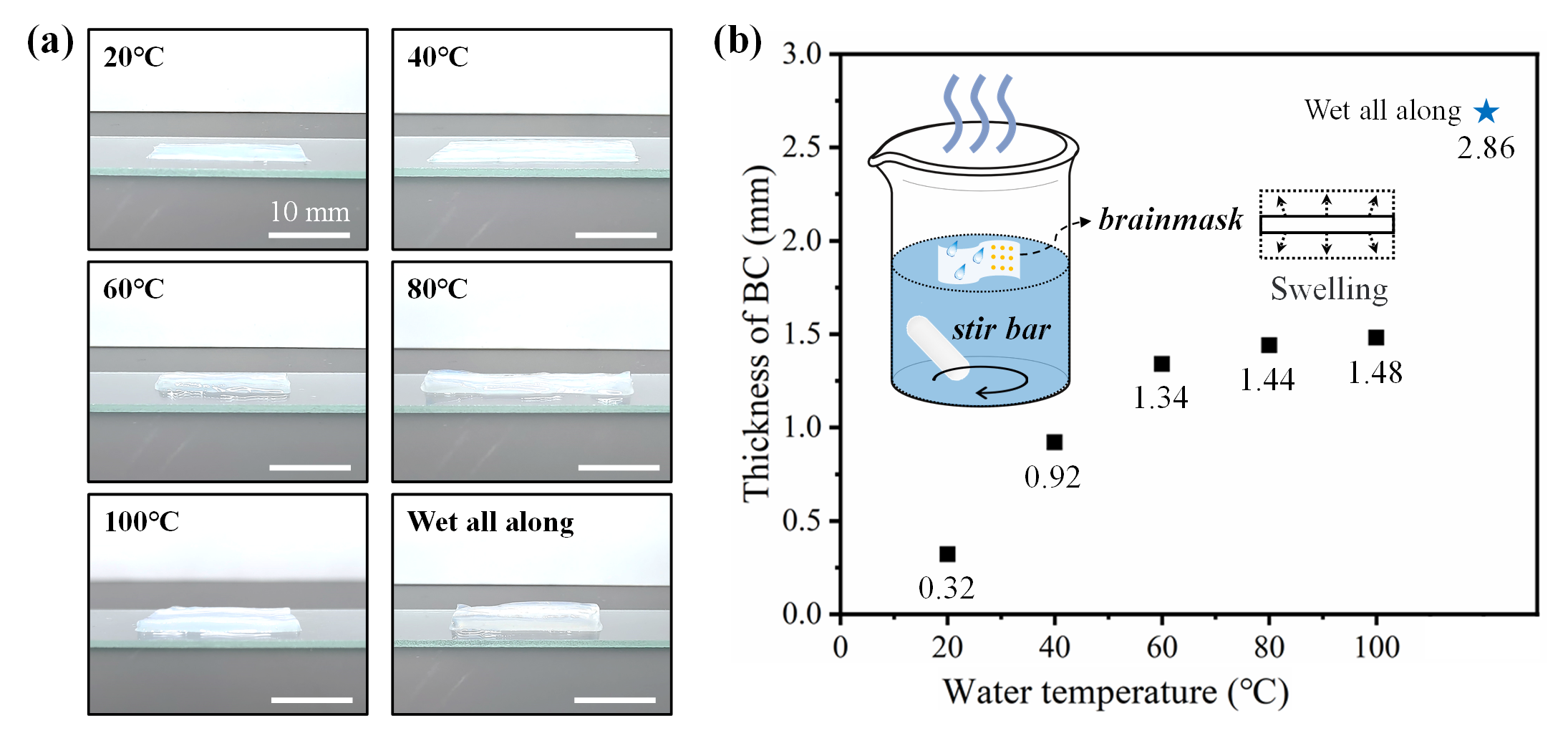


**Fig. S8** Thickness of BC in water absorption test. (**a**) Photos and (**b**) thickness values of dry BC immersed in deionized water with temperature from 20℃ to 100℃ and wet BC all along.


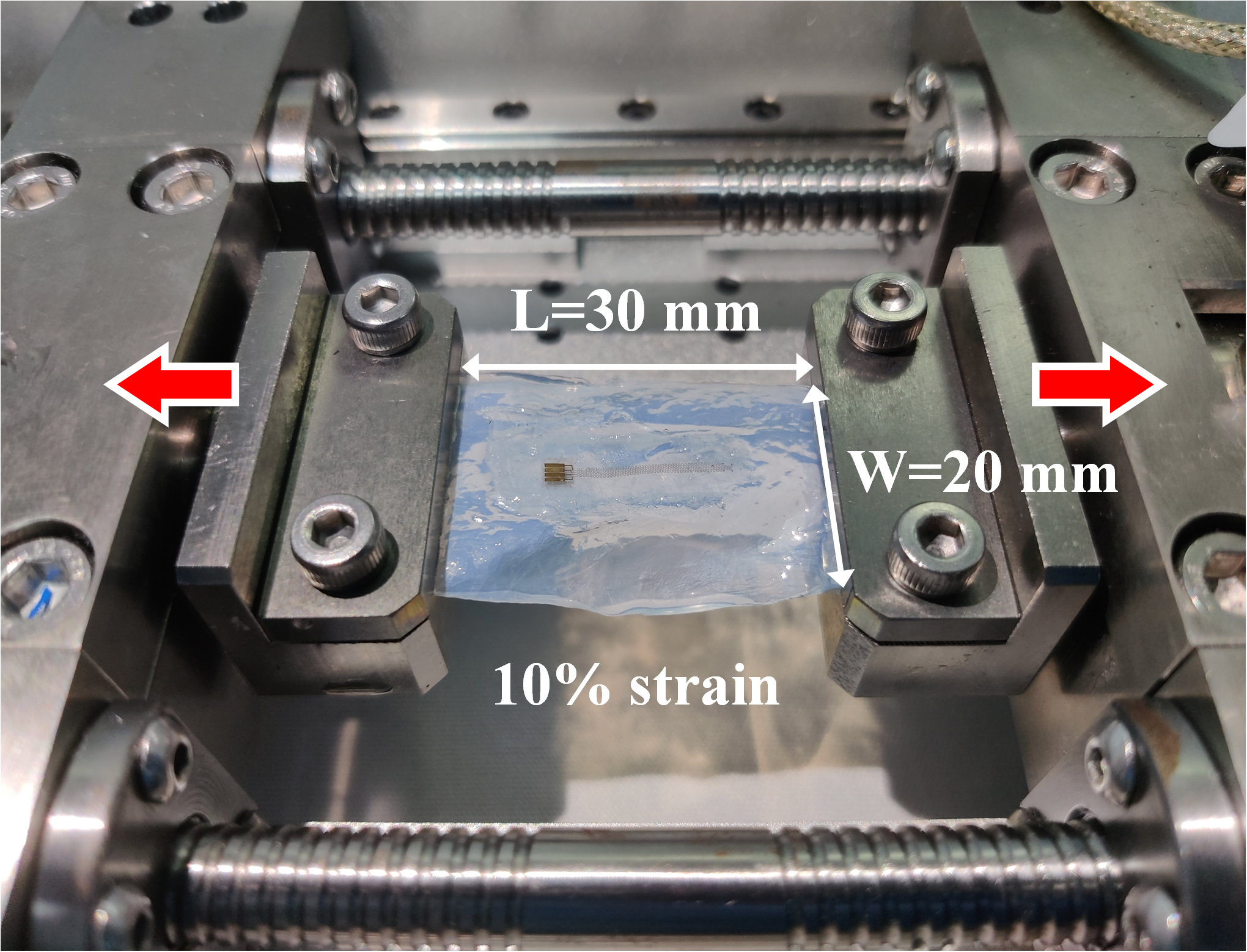


**Fig. S9** The wet Brainmask sample on the in-situ mechanical tensile test system for cyclic tensile at 10% strain.


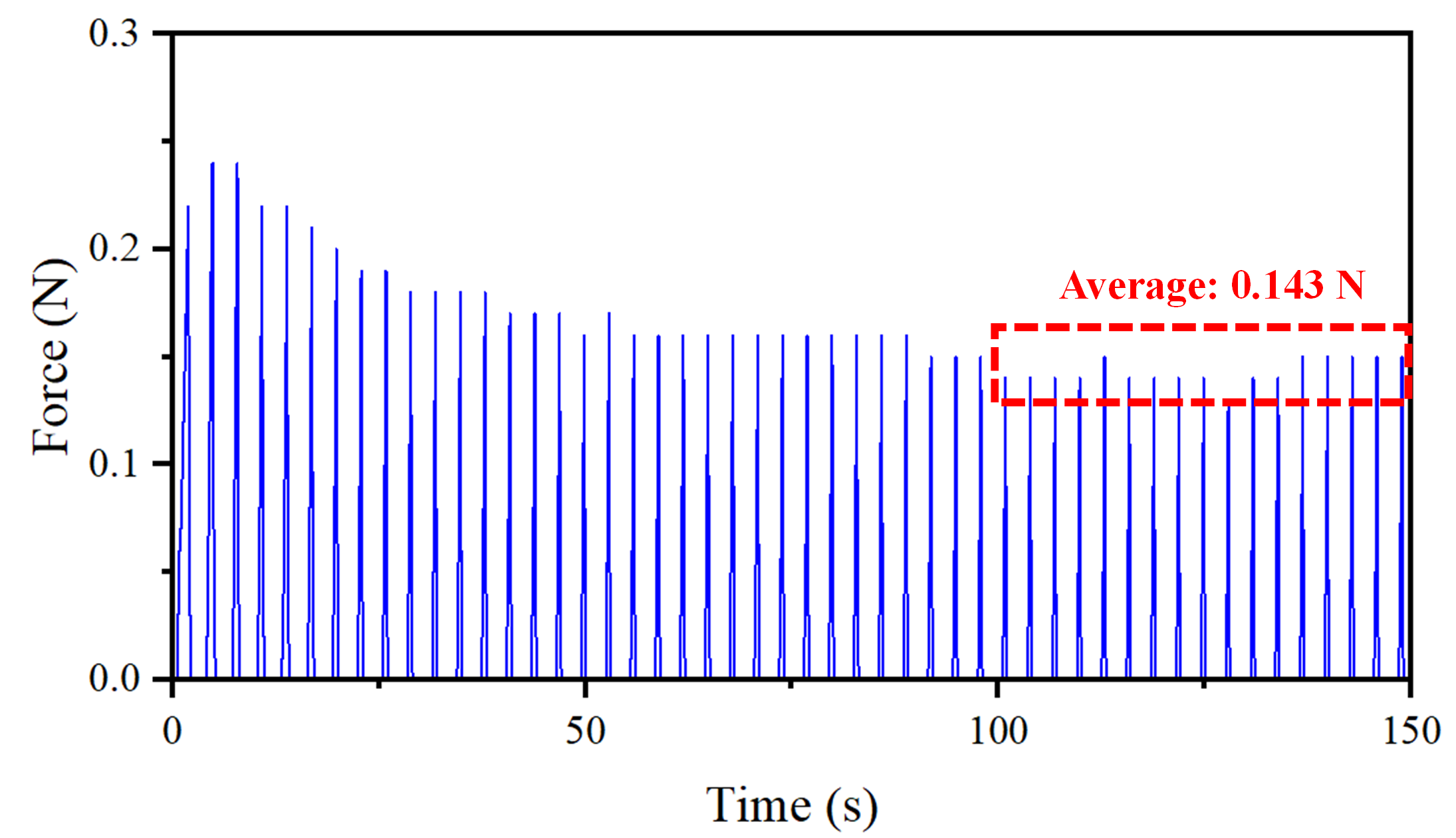


**Fig. S10** The measured tensile force of the wet Brainmask sample with 20 cycles/min.


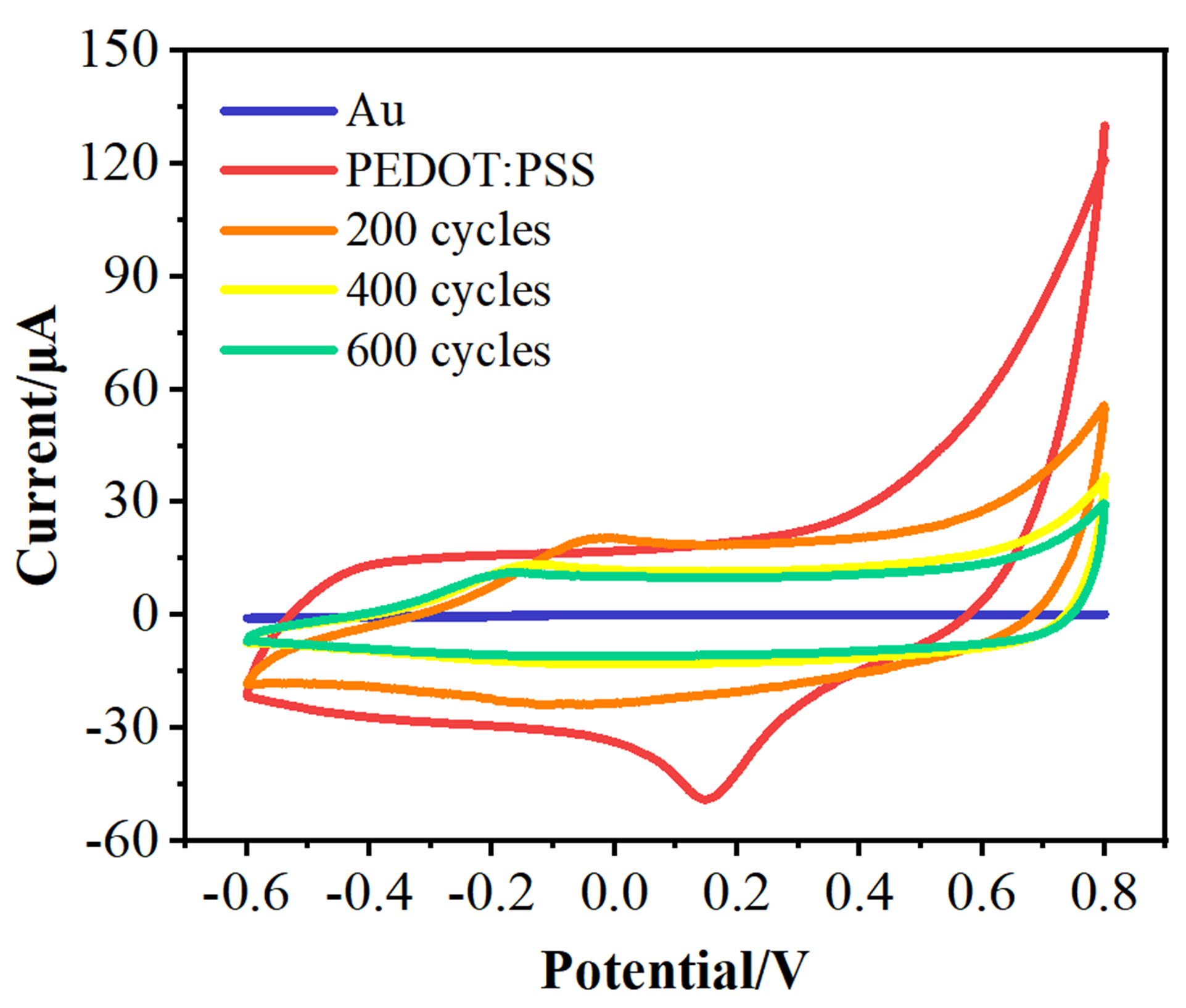


**Fig. S11** CV curves of bare gold, PEDOT:PSS modification, as well as 200, 400 and 600 cycles of CV scanning in the ultrasonic bath (100 W, 40 kHz).


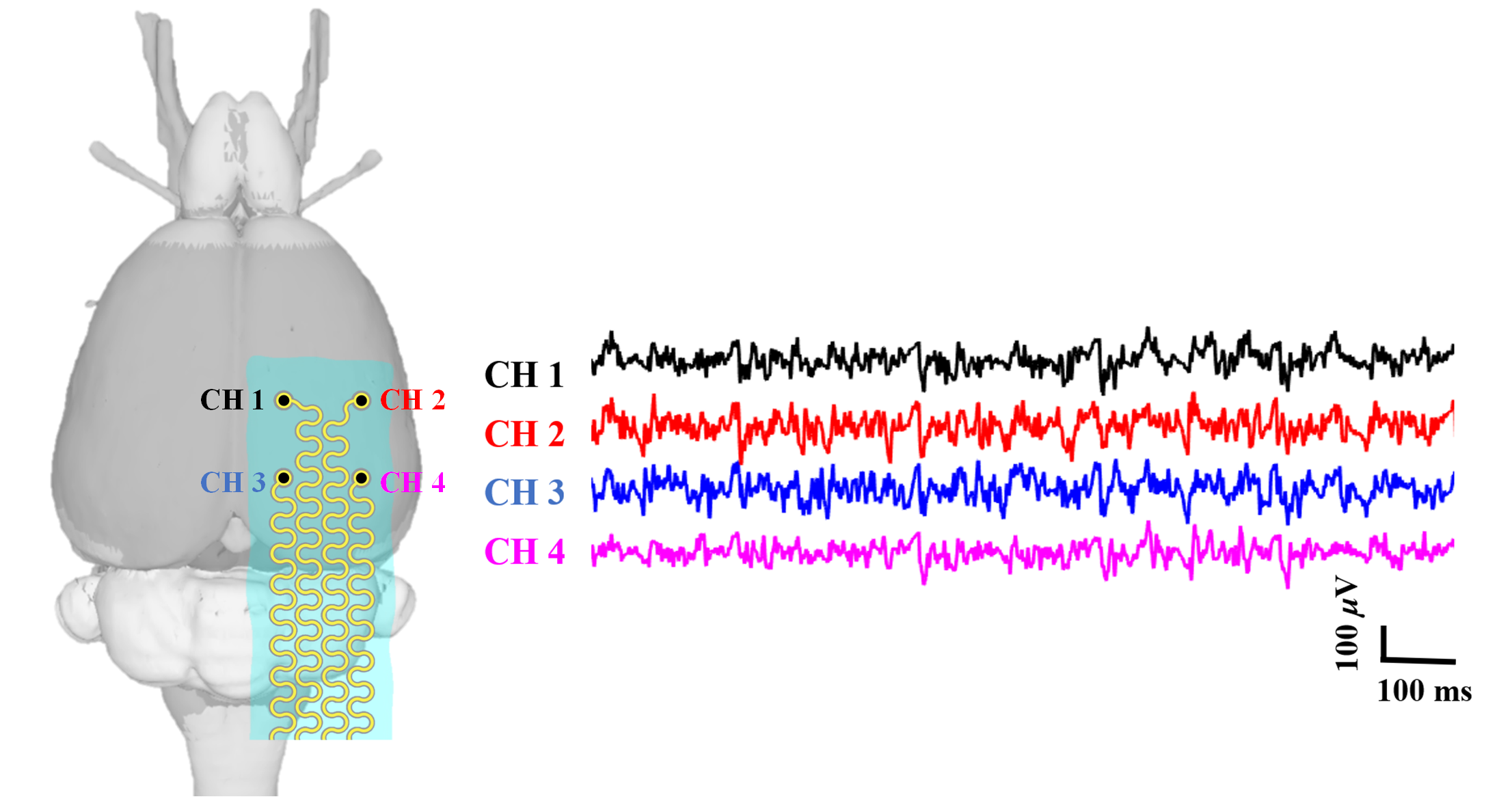


**Fig. S12** Illustration of the recording position and the ECoG signals of the 4-channel Brainmask device.

**References:**

[s1] Sauter-Starace, F., et al. Long-term sheep implantation of WIMAGINE®, a wireless 64-channel electrocorticogram recorder. *Front. Neurosci.* **13**, 847 (2019).

[s2] Larzabal C. et al. Long-term stability of the chronic epidural wireless recorder WIMAGINE in tetraplegic patients. *J. Neural Eng.*, **18**, 056026 (2021).
